# Benefit from surgery with additional radiotherapy in N1 head and neck cancer at the time of IMRT: A population-based study on recent developments

**DOI:** 10.1371/journal.pone.0229266

**Published:** 2020-02-26

**Authors:** Christoph Evers, Christian Ostheimer, Frank Sieker, Dirk Vordermark, Daniel Medenwald

**Affiliations:** 1 Medical Faculty, Martin Luther University Halle-Wittenberg, Halle (Saale), Saxony-Anhalt, Germany; 2 Department of Radiation Oncology, University Hospital Leipzig, Leipzig, Saxony, Germany; 3 Department of Radiation Oncology, Martin Luther University Halle-Wittenberg, Halle (Saale), Saxony-Anhalt, Germany; 4 Institute of Medical Epidemiology, Biostatistics and Informatics, Martin Luther University Halle-Wittenberg, Halle (Saale), Saxony-Anhalt, Germany; Chang Gung Memorial Hospital at Linkou, TAIWAN

## Abstract

**Background:**

Currently, the role of adjuvant irradiation in head and neck cancer (HNC) patients with N1-lymph node status is not clarified.

**Objectives:**

To assess the population-based effect of recent developments in radiotherapy such as intensity-modulated radiotherapy (IMRT) in relation to overall survival (OS) together with surgery in N1 HNC patients.

**Materials and methods:**

We used 9,318 HNC cases with pT1/2 N0/1 disease from German cancer registries. Time of diagnosis ranged from January 2000 to December 2014, which we divided into three periods: (low [LIA] vs intermediate [IA] vs high [HIA] IMRT availability period) based on usage of IMRT in Germany. For each period, we examined a possible association between treatment (surgery vs. surgery and radiotherapy) in terms of OS. Statistical analyses included Kaplan–Meier and multivariate Cox regression (models adjusted for HPV-related cancer site).

**Results:**

Temporal analysis revealed increasing usage of IMRT in Germany. In patients with N1 tumours, a comparison of patients treated with and without radiotherapy during the HIA period showed a superiority of the combined treatment as opposed to surgery alone (HR 0.54, 95%CI: 0.35–0.85, p = 0.003). The survival analyses related to treatments in terms of period underlined the superiority of surgery plus radiotherapy between periods IA and HIA (p = 0.03).

**Conclusion:**

The advent of IMRT, additional radiotherapy may present a survival advantage in patients with N1 HNC when combined with surgery.

## Introduction

The incidence of head and neck cancer (HNC) reaches approximately 4–5/100,000 inhabitants per year, and it is twice as prevalent among men compared to women.[1] In recent decades, increases in the incidence were mostly confined to women, with a rise in the incidence of papilloma virus (HPV) infections contributing.[1, 2]

Cervical positive lymph nodes represent a key prognostic factor in HNC which is most commonly related to recurrence.[3] Post-operative radiotherapy (PORT) in patients with high-risk features (such as perineural invasion [PNI] or lymphovascular space invasion [LVSI]; see National Comprehensive Cancer Network [NCCN] guidelines) is usually conducted to reduce the risk of recurrence. Retrospective studies have demonstrated a lower risk of recurrence and an improved OS for PORT in high-risk situations.[4–7] There is some debate regarding the effectiveness of post-operative radiotherapy in head and neck tumours in localised stages (T1/2) with solitary affected lymph nodes (N1).[8]

Regarding the question of PORT in T1/2 HNC, the NCCN guidelines provide the following recommendation in relation to risk profile: Adjuvant irradiation should be used in patients with oropharynx and hypopharynx cancer and a pN0 or N1 situation as soon as multiple minor risk factors—e.g., perineural invasion, lymphovascular space invasion or close margins (< 5 mm)—appear.[9, 10] If an extranodal extension (ECE) and/or positive resection margins occur, adjuvant chemoradiotherapy is recommended.[9–11] In patients with oral cavity cancer and a N1 situation, adjuvant irradiation should be considered.[11]

In recent years, the more advanced technique of intensity-modulated radiotherapy (IMRT) entered clinical practice.[12] It improved the protection of organs at risk, enabling delivery of a higher dose, and thus improving health outcomes.[13]

Based on current evidence, the role of adjuvant irradiation in patients with N1-lymph node status is not yet clarified. In addition, most of the present studies consider time periods (1984–2009) during which the use of IMRT was not yet widely used.[8] The study’s objective was to estimate the temporal change of overall survivals of N1 cases in the context of treatment combining surgery and radiotherapy in a population-based setting.

## Materials and methods

### Data set and subgroup formation

Our retrospective cohort study is based on data provided by the German Centre for Cancer Registry Data (GCCR) of the Robert Koch Institute. After a quality check by the GCCR, the regional centre registry data are pooled into a national epidemiological data set, which is issued for subsequent analysis.[14] Our study covers all patients with oral cavity, floor of the mouth, oral tongue, palate, oro- and hypopharynx carcinoma diagnosed between 2000 and 2014. Identification is based on the recent version of the *International Statistical Classification of Diseases and Related Health Problems German Modification* (ICD-10-GM).[15] The following ICD-10 codes were included: C02, C04–C06, C10, and C12–C14. For subsequent analyses, only cases with available histopathological grading were considered. Though the cancer registry reports “cases”, we used the terms “patient” and “cases” as synonyms.

In order to define time periods, data from the Research Data Centre (RDC) of the Federal Statistical Office and states’ Statistical Offices were used. These data include all inpatient cases in Germany, based on insurance claims and incorporating information about the German Modification of the International Classification of Procedures in Medicine (OPS) and the underlying disease.[16] IMRT (OPS: 8–522.9) was recorded as a procedure since 2008 in diagnosis-related groups’ (DRG) statistics as a subset of procedures encoding high-voltage radiotherapy (OPS: 8-522.x). For the definition of time periods, cases with a diagnosis of HNC recorded in this data set were used. Cases treated with IMRT (at least one fraction as IMRT) were related to all high-voltage radiotherapy procedures on a monthly basis (S1 Fig).[17] Based on these data, we can distinguish three periods: years before the widespread introduction of IMRT (< 2008, usage: ≤ 20%), interim period (2008–2010, usage: 20–50%) and the IMRT era, starting in 2011, with IMRT applied in more than 50% of treated cases throughout the year.

Histological inclusion criteria were epithelial, squamous cell, basal cell, transitional and adenocarcinomas. Classification is based on *International Classification of Diseases for Oncology*, *Third Edition* (ICD-O-3).[18] The following codes were included: 8001–8009, 8010–8049, 8050–8089, 8090–8119, 8120–8138, and 8140–8380. In German registries TNM was either marked as ‘‘clinical” (cT/N) or ‘‘pathological” (pT/N).[19] In the data, information on treatment refers to the application of surgery and/or radiotherapy. However in the manuscript, we presume that radiotherapy is delivered after surgery.

As the quality of each register differs depending on the respective German state, only high-quality registries from the federal states of Schleswig–Holstein, Rhineland–Palatinate, Bavaria, Mecklenburg–Vorpommern, Brandenburg, Berlin, Saxony–Anhalt, Saxony and Thuringia were included.[19] High-quality named registries included data on diagnosis and basic treatment since 2004 and sufficient data for risk stratification by tumour stage and grading for at least 70% of patients.

**Fig 1:**
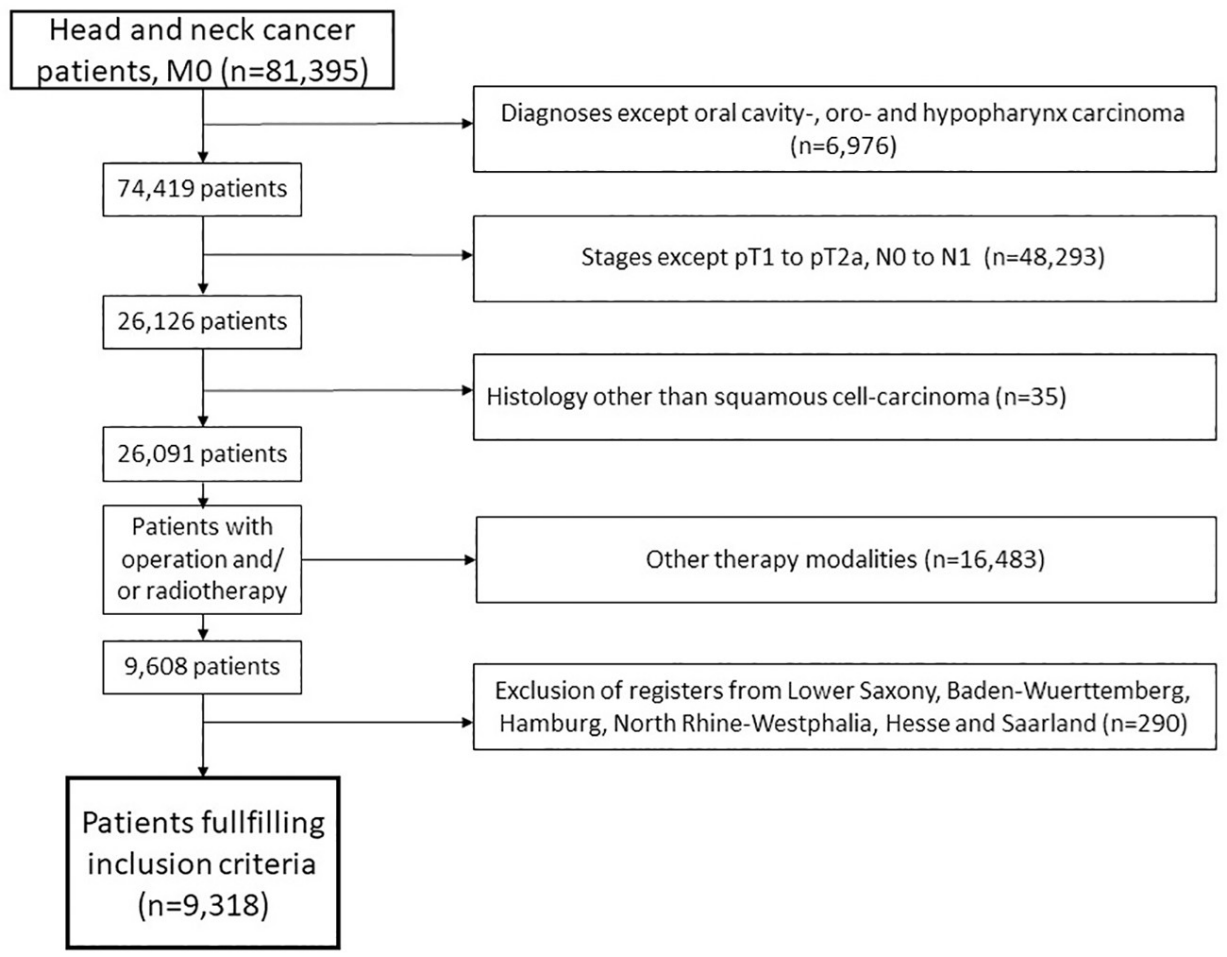
Quorum flowchart of the reasons for non-participation. The GCCR register contained, at the time of the query, 81,395 non-metastasised head and neck cancer patients.

We only included non-metastasised (M0) tumours in stage pT1 or pT2 and N0 or N1. Other inclusion criteria are depicted in Fig 1.

Staging was performed according to the TNM classification of malignant tumours. Therefore, the sixth[20] and seventh[21] edition of the American Joint Committee on Cancer staging guidelines were used. No relevant changes were made between the sixth and seventh edition regarding T1 and T2 stages of HNC.

To distinguish between HPV-positive and HPV-negative cases, HPV-associated sites were defined, according to Jansen et al. and Chaturvedi et al., as the base of the tongue (International Classification of Disease for Oncology version-3 [ICD-0-3] topography code: C019), lingual tonsil (C024), palatine tonsil (C090–099), oropharynx (C100–109), and Waldeyer’s ring (C142).[22] The HPV-unrelated sites included cancers of the tongue (C020–023 and C025–029), gum (C030–039), floor of the mouth (C040–049), palate (C050–059), and parts of the mouth (C060–069). Likewise, we excluded cancers of the nasopharynx, salivary glands and larynx.[22, 23]

As a primary endpoint, we focused on overall survival after the month of diagnosis. Patients were censored at the last date they were known to be alive or December 31, 2014 –whichever came first. All tumours diagnosed during autopsy or based on a death certificate were excluded.

As data were provided by the GCCR, no approval by an ethics committee was required to conduct the analyses (terms outlined by the Robert Koch-Institut apply[24]).

### Statistical analysis

Baseline differences between treatment eras and treatment groups (surgery, RT) were compared using mean values and 95% confidence intervals. Survival analyses were censored after month 60. For analyses of overall survival, we used Cox proportional hazard regression models comparing time periods as outlined above: “high IMRT availability period” (HIA) versus “intermediate IMRT availability period” (IA) and “low IMRT availability period” (LIA). In the Cox regression analyses, we adjusted for the following potential confounders: age at diagnosis, grading, T-stage, N-stage, sex, and HPV-status defined by site (see above). For graphical illustrations, we plotted Kaplan–Meier curves. In order to avoid biases due to different case numbers between periods, all cases were included in one Cox regression model adding a period*treatment interaction term. This term estimates, statistically, possible differences in hazards related to treatment between periods. Thus, based on the assumption of proportional hazards, as required by Cox regression models, population differences between time periods related to treatments can be compared directly in one model. This method can estimate effect more accurately compared to methods such as propensity score matching, which might suffer from insufficient statistical power and confounder adjustment. We compared the treatment effects of each period in relation to the remaining two. All statistical tests were two-sided, with a threshold of p ≤ 0.05 for statistical significance. We used SAS^®^, Version 9.3 (SAS Inc., Cary, NC, USA) for statistical analyses.

## Results

### Study population

The query yielded 81,395 patients, with 9,318 patients remaining after exclusion of those who did not meet all inclusion criteria listed in Fig 1. Most of the exclusions (n = 48,293) were due to patients having tumours at clinical stages other than T1 to T2 and N0 to N1. Others had a diagnosed tumour identified through either autopsy or death certificate.

### Patients’ demographics

Patient and disease characteristics are summarised in Table 1. On average, patients who underwent surgery only were older (62.21 years; 95% CI 61.92–62.50 vs 58.64 years; 95% CI 58.26–59.03, p < 0.001) and had more differentiated tumours (76.84% vs 69.20%, p < 0.001) than patients who received surgery and radiotherapy. Median follow-up was 57.5 months for the whole group (87.3, 54.7, and 22.2 months for periods LIA, IA, and HIA, respectively).

**Table 1:** Patient and tumour characteristics of the entire, LIA, IA, and HIA patient cohort.

		All	LIA	%	IA	%	HIA	%	p-value
No.		9318	3987	42.79	2148	23.05	3183	34.16	
Age	Mean years	61.17	59.94	-	60.68	-	63.05	-	<0.001
Sex	Male	6720	2977	74.67	1540	71.69	2203	69.21	<0.001
	Female	2598	1010	25.33	608	28.31	980	30.79	
Subsite	Oral cavity	8234	3464	86.88	1924	89.57	2846	89.41	<0.001
	Oropharynx	637	301	7.55	134	6.24	202	6.35	
	Hypopharynx	447	222	5.57	90	4.19	135	4.24	
Histology	SCC	9078	3882	97.37	2092	97.39	3104	97.57	<0.001
	Other	235	102	2.56	55	2.56	78	2.45	
	Unknown	5	3	0.08	1	0.05	1	0.03	
Grading	G1-2	6953	2949	73.97	1600	74.49	2404	75.53	<0.001
	G3-4	1710	751	18.84	425	19.79	534	16.78	
	Unknown	655	287	7.20	123	5.73	245	7.70	
T-stage	1	5331	2229	55.91	1194	55.59	1908	59.94	<0.001
	2	3987	1758	44.09	954	44.41	1275	40.06	
N-stage	0	7659	3169	79.48	1792	83.43	2698	84.76	<0.001
	1	1659	818	20.52	356	16.57	485	15.24	
Therapy	PORT	2711	1359	34.09	609	28.35	743	23.34	<0.001
	No PORT	6607	2628	65.91	1539	71.65	2440	76.66	

*LIA* = low IMRT availability period; *IA* = intermediate IMRT availability period; *HIA* = high IMRT availability period; *No*. = Number; *SCC*. = s*quamous cell carcinoma*; *G* = grading, *PORT* = Post-operative radiotherapy. P-value from Mann-Whitney U (metric variables) and Chi^2^ test (frequencies).

### Survival analyses

#### Patients with N0

**Fig 2:**
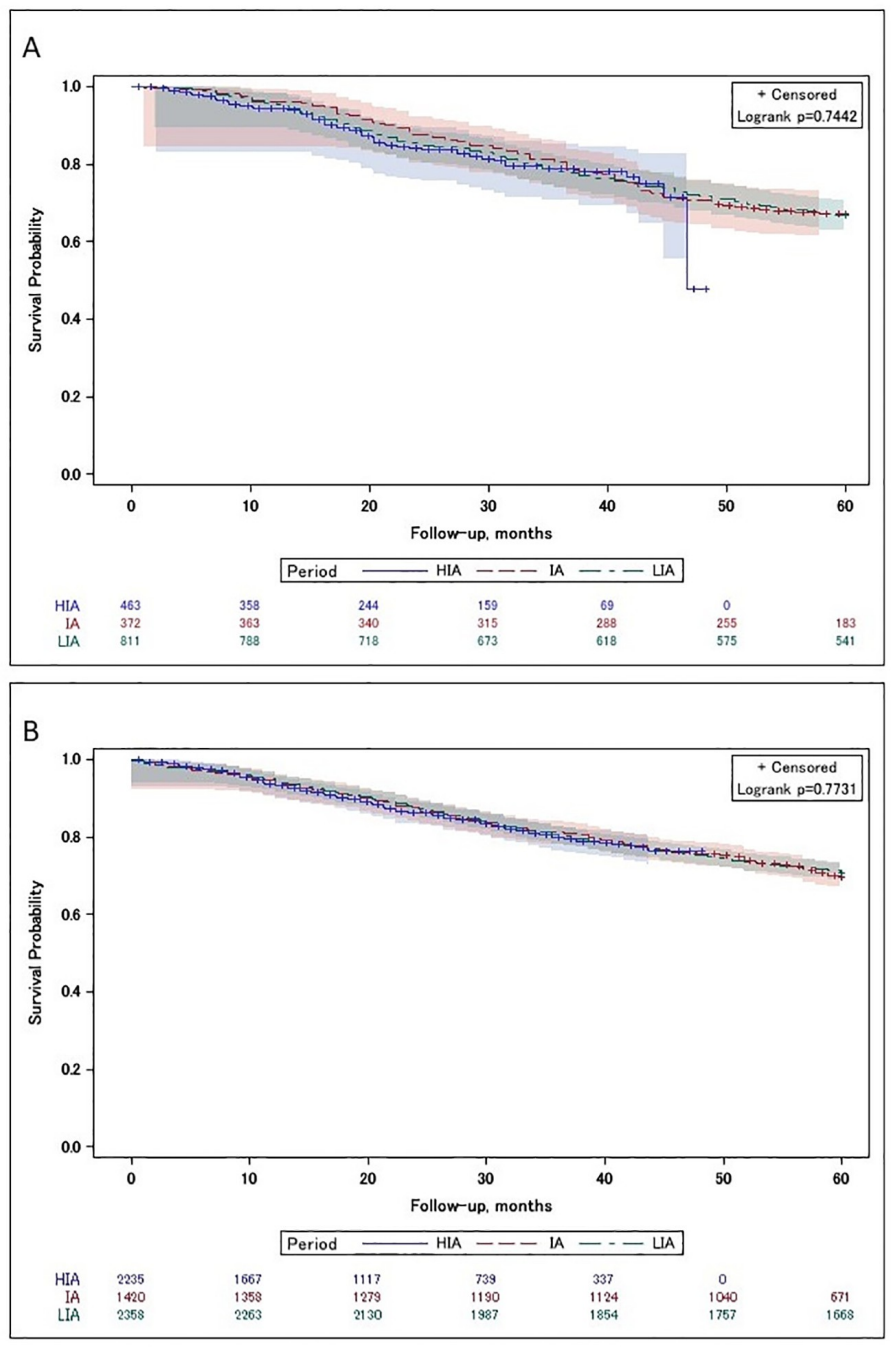
Kaplan–Meier survival curves with number of subjects at risk and 95% Hall–Wellner bands for the (A) No PORT and (B) PORT study population in a pN0 situation, related to the treatment periods. *IMRT* = intensity-modulated radiotherapy, *LIA* = low IMRT availability period, *IA* = intermediate IMRT availability period, *HIA* = high IMRT availability period.

A total of 7,659 patients in a N0 situation were considered in the Kaplan–Meier analyses. The overall survival was measured for both patients treated surgically without radiotherapy and patients who received surgery and radiotherapy, comparing the three time periods. The unadjusted Kaplan–Meier estimates of OS in each therapy group are shown in Fig 2. Median overall survival was not reached.

**Fig 3:**
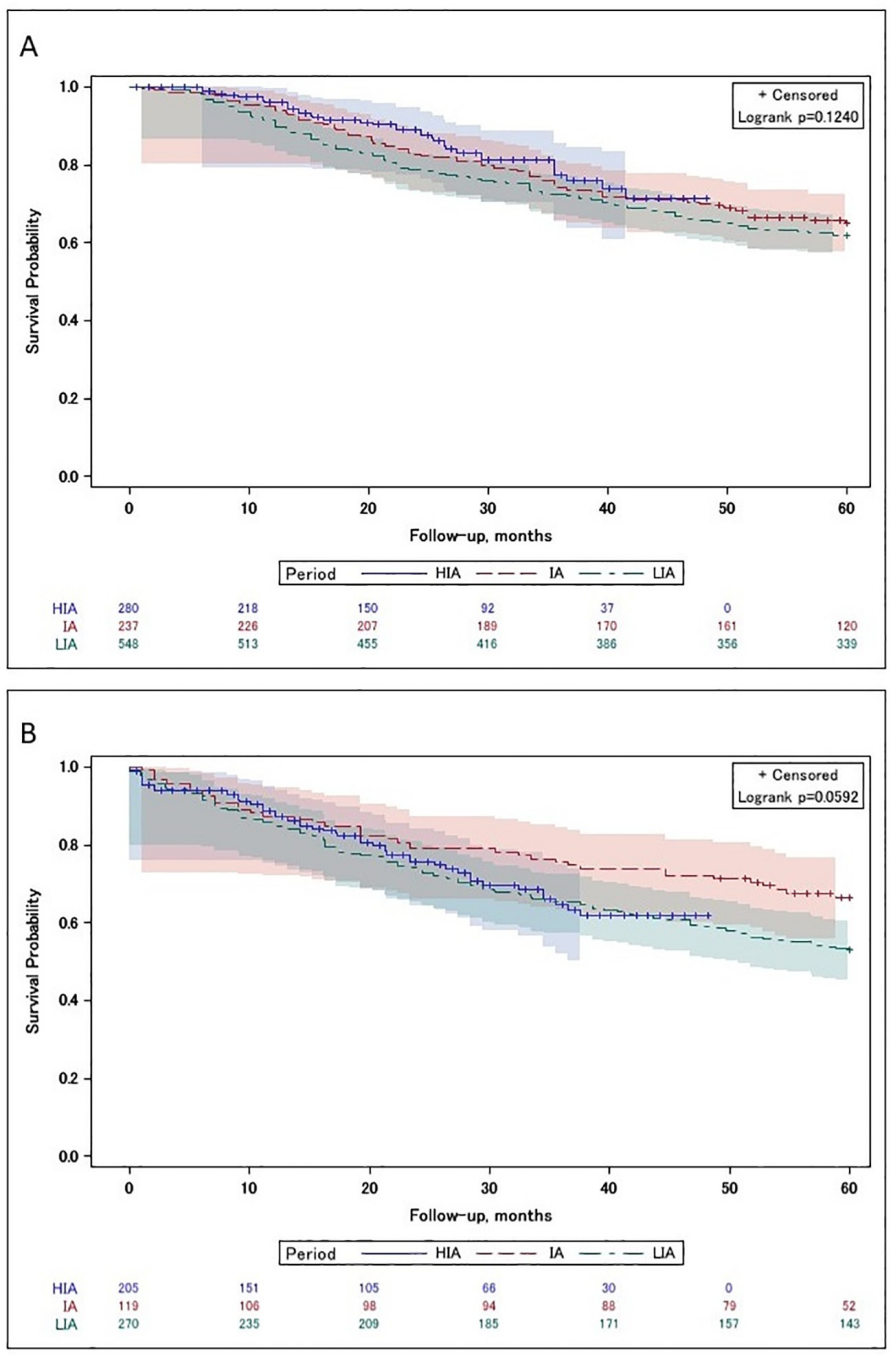
Kaplan–Meier survival curves with number of subjects at risk and 95% Hall–Wellner bands for the (A) No PORT and (B) PORT study population in a N1 situation, related to the treatment periods. *IMRT* = intensity-modulated radiotherapy, *LIA* = low IMRT availability period, *IA* = intermediate IMRT availability period, *HIA* = high IMRT availability period.

In patients with N0 tumours, a comparison of patients treated with and without radiotherapy within the LIA, IA, and HIA period indicated comparable mortality in both treatment regimens (LIA: HR 0.99, 95%CI: 0.87–1.13 and IA: HR: 1.03, 95%CI: 0.83–1.27 and HIA: HR: 1.03, 95%CI: 0.77–1.38) when confounders were considered (Fig 3A).

#### Patients with N1

**Fig 4:**
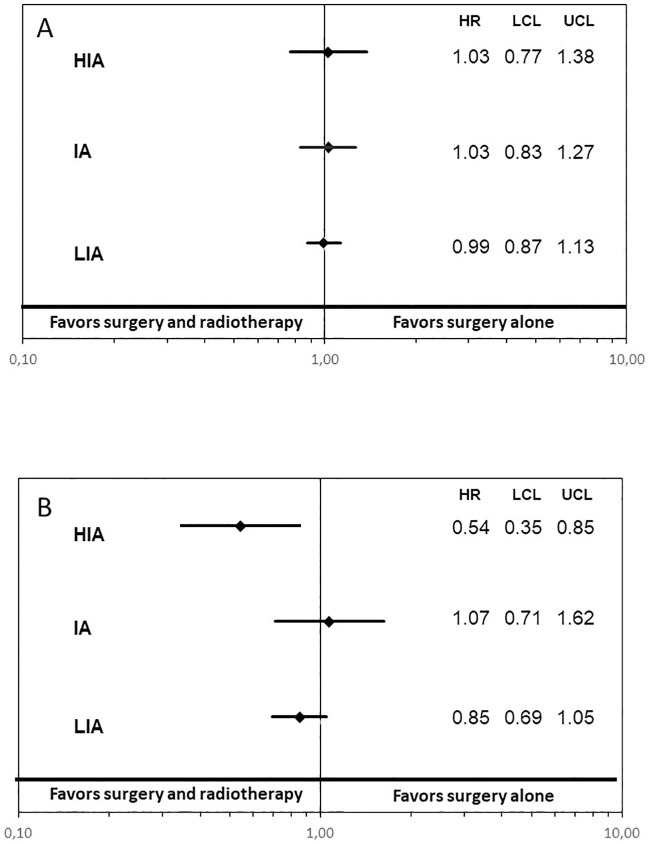
Impact of surgery with additional radiotherapy on mortality in the trend of interaction by treatment period in pN0 (A) and N1 (B) situations. *HR* = hazard ratio; *LCL* = lower confidence level; *UCL* = upper confidence level; *LIA* = low IMRT availability period; *IA* = intermediate IMRT availability period; *HIA* = high IMRT availability period.

A total of 1,659 patients in a N1 situation were considered in the Kaplan–Meier analyses. The overall survival was measured for both patients treated surgically without radiotherapy and patients who received surgery and radiotherapy, comparing the three time periods. The unadjusted Kaplan–Meier estimates of OS in each therapy group are shown in Fig 4. This analytical method indicated low evidence in favour of patients who underwent PORT in the HIA period (p = 0.06). Median overall survival was not reached.

In all patients with N1 tumours, a comparison of patients treated with and without additional radiotherapy within the LIA period failed to support evidence of a superiority of one of the above-mentioned treatment regimens in adjusted models (HR 0.85, 95%CI: 0.69–1.05). Likewise, in the comparison of irradiated and primary operated patients in a N1 situation within the IA period there was no benefit of either treatment option (HR 1.07 95%CI: 0.71–1.62). Analysis within the HIA, however, revealed evidence for a superiority of a combined treatment as opposed to surgery alone (HR 0.54, 95%CI: 0.35–0.85, p = 0.003) (Fig 3B).

In the subgroup analysis, we found that the effect was primarily connected to cancer in the oral cavity. For patients with N1 oral-cavity tumours, a comparison between patients treated with and without radiotherapy within the LIA period showed no evidence for a better survival prospect of either treatment option (HR 0.84, 95%; CI: 0.66–1.06) after confounder adjustment. Likewise, the comparison between irradiated and primary-operated oral-cavity cancer patients in an N1 situation within the IA period revealed comparable effects (HR 1.09, 95%; CI: 0.70–1.72). Analysis within the HIA, however, showed the statistically-significant superiority of combined treatment compared to surgery alone (HR 0.59, 95%; CI: 0.36–0.95, p = 0.013). Although cancers in the hypopharynx might contribute substantially, effect estimates showed wide confidence intervals.

In the analysis of possible differences in hazards related to treatment between periods, all N1 cases of all three periods were included. In patients with N1 tumours, a comparison of patients treated with and without additional radiotherapy showed no evidence for an interaction between the LIA and HIA periods (p = 0.074). However, the same analytical method indicated a significant interaction between the IA and HIA periods (p = 0.029), revealing evidence for a changing treatment effect in preference of a bimodal treatment.

#### Sensitivity analyses

Relevant changes to effect estimates were produced by considering every case rather than only those from high-quality registries. A comparison of the multivariate analyses of every HNC patient in N1 stage T1 or T2 found no major difference for the HIA period between analyses from all registries (HR 0.57, 95%; CI: 0.36–0.89) and those from high-quality registries (HR 0.54, 95%; CI: 0.35–0.85). Similarly, comparison of the multivariate analyses for every oral-cavity cancer patient in N1 stage T1 or T2 during the HIA period found no major difference between those from all registries (HR 0.61, 95%; CI: 0.38–0.98) and those from high-quality registries (HR 0.59, 95%; CI: 0.36–0.95).

## Discussion

Our analysis provides evidence for a superiority of surgery plus radiotherapy as compared to surgery alone in patients with pT1/2 N1 HNC within the HIA period. The analysis of possible differences in hazards related to treatment between periods showed some evidence for a changing treatment effect, as indicated in a significant interaction between the IA and HIA periods. Analysis regarding pN0 patients served as a control, with no evidence for changes in the treatment effect between periods.

To our knowledge, this study constitutes the first analysis of post-treatment mortality in early-stage HNC patients related to radiotherapy techniques in a population-based setting. In this study, we based the definition of time periods on the application of IMRT in an inpatient setting covering the whole of Germany. Thus, the definition of time periods is likely to reflect the application of IMRT in HNC in Germany in general, avoiding bias due to an arbitrary period definition. Focus was put on intensity-modulated irradiation technologies, with which normal tissue can be better spared than with older technologies. Several publications were able to indicate a reduction of side effects when IMRT is applied.[25, 26]

A meta-analysis by Moergel et al. on N1 neck cancer with small oropharyngeal squamous cell carcinoma (SCC) identified seven studies.[8] In this analysis, however, only two out of the seven considered studies compared PORT as an adjuvant treatment to surgery alone. Due to poor model quality, the meta-analysis failed to present a clear result.[8] As most of the studies were mostly conducted in the era before the widespread introduction of IMRT (1984–2009), an evaluation of the influence of radiotherapy techniques is difficult. The same applies to the publications by Ang et al.,[27] Jäckel et al.,[4] Kao et al.[5] and Schmitz et al.[28] These studies were published between 2001 and 2009. The prospective study by Ang et al. did not find a benefit for PORT in a N1 situation when patients were free of adverse features. Jäckel et al. examined N1 neck cancers without ECE where most of the patients had selective neck dissection. Isolated nodal failure (surgery 10% vs PORT 2%), all nodal failures (21% vs 9%). Three-year neck recurrence rate improved with PORT (11% vs 3%). The data suggested a trend towards improved regional control for N1 with PORT. Kao et al. could not detect a difference between patients with oral cavity T1/2 N1 cancer with and without PORT regarding 5-year OS (38.7% vs 36.0%; p = 0.23). The same study, however, examined oropharyngeal carcinomas at the same tumour stage, revealing differences between patients with and without adjuvant radiotherapy. Five-year OS stood at 67.9% vs 65.0%; p = 0.003. Schmitz et al. state a N1 failure rate without PORT of 9% versus with PORT of 5%, but concluded that additional radiotherapy would only be justified for pN2b and ECE.

A recent retrospective analysis from 2016 by Chen et al. used OS as the primary endpoint.[29] Here, a distinction was made between pT1 and pT2, and N1 and pN2, as well as patients under and over the age of 70. The study concludes that adjuvant radiotherapy may be associated with improved survival in patients with N1 oral cavity (58.8% vs 54.4%; p = 0.007) and oropharyngeal SCC (85.1% vs 74.7%; p < 0.001), especially in those younger than 70 years or those with pT2 disease. Despite this recently published study lacking information covering radiotherapy techniques and confounding HPV, it shows that PORT is associated with improved OS.

Quality of life is also affected by IMRT. The effect was measured using the EORTC questionnaire QLQ-C30. Significant differences between IMRT and the 3D-conformal radiotherapy technique were found in: emotional functioning (98:86, p = 0.008), role functioning (98:86, p = 0.008) and dry mouth 33:48 (p = 0.05) after 12 months.[26]

In our population-based study, N1 HNC patients with radiotherapy and surgery had a survival benefit as compared to those without, but only in a time period when IMRT was applied to the majority of patients. This might have been due to the reduction of toxicities that lead to life-threatening changes, such as (1) fewer dose gaps and fewer high dose levels in terms of sufficient homogenous dose coverage in recent times, (2) better regeneration of organs at risk, (3) fewer mucositides, (4) less osteoradionecrosis, (5) better nutritional situation, (6) fewer infections, and (7) shorter hospitalisation.[30, 31]

The effect of a higher irradiation dose in the context of IMRT is most consequential when radiation is delivered as a simultaneous integrated boost (SIB). Advantages include moderate treatment acceleration, reduced treatment time, and the possibility of dose escalation in the gross tumour volume.[32–34] Given it is probable that few cases are treated with SIB in a post-operative setting, this might play a less important role in our study. Instead, the most substantial contributors to the survival benefit found by this study were likely the fewer toxicities resulting from IMRT, a lower integral dose and the consequent sparing of at-risk organs.[34] In the PARSPORT trial, compared with 3D-conformal radiotherapy technique, using IMRT produced significant reduction of Grade 2 xerostomia at 12 months and 24 months (36% and 54%). In both cases, recovery of saliva production was achieved faster, with a consequent improvement in quality of life.[35] Thus, it is presumed that improved survival results from lower toxicity rather than loco-regional control.

In the subgroup analysis, cancer of the oral cavity contributed most significantly to findings. Yao et al. reported similar findings in “Iowa experiences”: compared to oropharynx and larynx carcinomas, two-year loco-regional control of the oral cavity tumours was significantly poorer (98%, 85%, and 78%, respectively).[36] Furthermore, from a clinical perspective, cancers of the oral cavity present the worst prognosis as few cases are HPV associated and they are difficult to resect completely.

More current prospective studies are needed to evaluate the effect of the introduction of IMRT in the population.

### Limitations

In the cancer registries, data on each conducted staging investigation, comorbidities, performance status, type of surgery, specific RT details (i.e., exact start date, dose, fractionation, and technique) and HPV status are lacking. The latter is, however, decisive for an increasing incidence of HNC as well as mortality prediction.[37] To draw a distinction between HPV-positive and HPV-negative cases, a selection as proposed by the publications by Jansen et al. and Pagedar et al. was made.[23, 38]

Our analyses demonstrated that the influence of HPV related location on overall survival did not change over time. Chaturvedi et al. published an analysis that showed an increase in incidence of oropharyngeal cancer in the United States since 1984 caused by HPV infections.[39] Stein et al. showed a significant increase in HPV-positive oropharyngeal cancers in Europe since 1995.[40] However, as we adjusted our cox proportional hazard model for HPV-associated tumour locations, a serious bias of effect estimates is unlikely. Effect estimates might only be biased if the proportion of HPV-positive cases, with a better survival prospect, increased in patients treated with radiotherapy and surgery and was strongest in the most recent period. However, this seems unlikely, as treatment de-escalation is preferred in HPV-positive cases. Thus, period effects might even be stronger than estimated in our study.

This study’s results are largely not subject to unmeasurable confounding factors with prominent effects on radiotherapy. These external factors, such as socioeconomic status and hospital volumes, are likely to influence both surgery and radiotherapy.[41, 42] For this reason, they are unlikely to bias estimates reported in this study because HNC is mostly treated in high-volume centres. In addition, HPV, being related to socioeconomic status, is an important survival predictor.[43] The study adjusted for HPV-related cancer sites, enabled the confounding effect of socioeconomic status to be taken into account. Due to a lack of information, the question of potential influence of R-status or risk factors (PNI and LVSI) also remains unanswered. The data set also does not contain details on locoregional recurrence. In the case of positive resection margins, chemotherapy should be applied in accordance with recent guidelines.[9, 10] In this study, all patients receiving chemotherapy were excluded (Fig 1). Thus, we do not expect a serious bias due to positive resection margins. Again, it seems unlikely that the effect of positive margins improved over time when no additional treatment (e.g., chemotherapy) was applied. A similar reason applies to the PNI which is a predictive factor of poor outcome in HNC.[44] The incidence of PNI in HNC has been reported to be 7.5–52%. A figure of 90% was reported in a necropsy series.[45]

## Conclusions

Our study shows that the impact of additional radiotherapy on survival has changed towards a survival benefit of a combined treatment after the widespread introduction of IMRT. With the advent of IMRT, this regime may present a survival advantage in patients with N1 HNC when combined with surgery. In light of our findings, further studies are needed to clarify the value of PORT in the era of IMRT.

## Supporting information

S1 Fig Development of the proportion of inpatients treated with IMRT in Germany. *IMRT* = Intensity-modulated radiotherapy; Identification of the respective periods by *; LIA: IMRT not refundable (from 2000 to 2007); IA: Annual mean of cases treated with IMRT in hospitalized patients <50%; HIA Annual mean of cases treated with IMRT in hospitalized patients > 50%.F.
(ZIP)
