# Supplementary material for: Benefit from surgery with additional radiotherapy in N1 head and neck cancer at the time of IMRT: A population-based study on recent developments
Source: PLoS One. 2020 Feb 26;15(2):e0229266. doi: 10.1371/journal.pone.0229266 (PMC7043743; doi:10.1371/journal.pone.0229266)
Supplement: S1 Fig — IMRT = Intensity-modulated radiotherapy; Identification of the respective periods by *; LIA: IMRT not refundable (from 2000 to 2007); IA: Annual mean of cases treated with IMRT in hospitalized patients <50%; HIA Annual mean of cases treated with IMRT in hospitalized patients > 50%.F. (ZIP) [file pone.0229266.s001.zip › S1.docx]

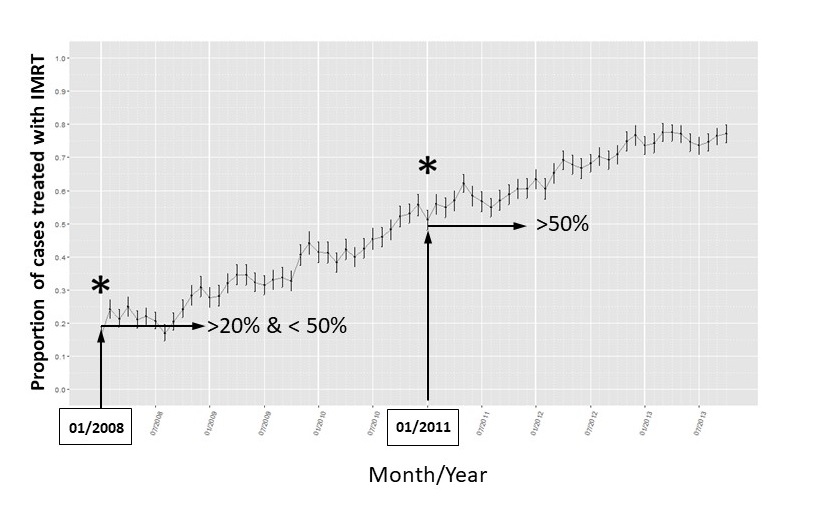


**S1 Fig. Development of the proportion of inpatients treated with IMRT in Germany**

*IMRT*= Intensity-modulated radiotherapy; Identification of the respective periods by *; LIA: IMRT not refundable (from 2000 to 2007); IA: Annual mean of cases treated with IMRT in hospitalized patients <50%; HIA Annual mean of cases treated with IMRT in hospitalized patients > 50%
